# Supplementary material for: Geographical distribution of two major quarantine fruit flies (Bactrocera minax Enderlein and Bactrocera dorsalis Hendel) in Sichuan Basin based on four SDMs
Source: PeerJ. 2024 Jan 8;12:e16745. doi: 10.7717/peerj.16745 (PMC10782948; doi:10.7717/peerj.16745)
Supplement: Supplemental Information 1 [file peerj-12-16745-s001.docx]

| **Species** | **longitude** | **latitude** |
| --- | --- | --- |
| Bactrocera minax | 110.2 | 24.816667 |
| Bactrocera minax | 111.45 | 25.35 |
| Bactrocera minax | 107.295883 | 25.91791 |
| Bactrocera minax | 108.405284 | 25.976409 |
| Bactrocera minax | 107.470556 | 26.09 |
| Bactrocera minax | 105.731459 | 26.30295 |
| Bactrocera minax | 105.121257 | 26.343215 |
| Bactrocera minax | 105.35 | 26.383333 |
| Bactrocera minax | 106.683671 | 26.410601 |
| Bactrocera minax | 110.05 | 26.616667 |
| Bactrocera minax | 109.692931 | 26.666344 |
| Bactrocera minax | 109.202621 | 26.679226 |
| Bactrocera minax | 107.1 | 26.683333 |
| Bactrocera minax | 109.248493 | 26.741504 |
| Bactrocera minax | 110.788875 | 27.012723 |
| Bactrocera minax | 110.366667 | 27.033333 |
| Bactrocera minax | 111.27601 | 27.088894 |
| Bactrocera minax | 111.246483 | 27.108199 |
| Bactrocera minax | 109.621635 | 27.372603 |
| Bactrocera minax | 110.2 | 27.45 |
| Bactrocera minax | 109.768987 | 27.459216 |
| Bactrocera minax | 109.687553 | 27.682645 |
| Bactrocera minax | 108.349771 | 27.742422 |
| Bactrocera minax | 108.193255 | 27.745029 |
| Bactrocera minax | 105.873215 | 27.755927 |
| Bactrocera minax | 104.593979 | 27.773173 |
| Bactrocera minax | 109.743902 | 27.792693 |
| Bactrocera minax | 104.534224 | 27.796515 |
| Bactrocera minax | 109.830858 | 27.809601 |
| Bactrocera minax | 109.702997 | 27.816668 |
| Bactrocera minax | 109.681217 | 27.850028 |
| Bactrocera minax | 104.485515 | 27.855491 |
| Bactrocera minax | 110.2 | 27.866667 |
| Bactrocera minax | 109.809096 | 27.871781 |
| Bactrocera minax | 109.930013 | 27.889738 |
| Bactrocera minax | 109.666494 | 27.909914 |
| Bactrocera minax | 103.559062 | 27.998073 |
| Bactrocera minax | 103.508523 | 28.113076 |
| Bactrocera minax | 108.564474 | 28.151616 |
| Bactrocera minax | 113.089386 | 28.182346 |
| Bactrocera minax | 107.533333 | 28.216667 |
| Bactrocera minax | 108.487142 | 28.225529 |
| Bactrocera minax | 110.164664 | 28.268229 |
| Bactrocera minax | 108.034608 | 28.280928 |
| Bactrocera minax | 113.583333 | 28.3 |
| Bactrocera minax | 111.123431 | 28.331701 |
| Bactrocera minax | 113.55 | 28.383333 |
| Bactrocera minax | 104.05 | 28.383333 |
| Bactrocera minax | 109.011751 | 28.435434 |
| Bactrocera minax | 112.916667 | 28.45 |
| Bactrocera minax | 111.399226 | 28.466531 |
| Bactrocera minax | 104.476625 | 28.50735 |
| Bactrocera minax | 110.216667 | 28.516667 |
| Bactrocera minax | 108.403961 | 28.591623 |
| Bactrocera minax | 109.150273 | 28.736671 |
| Bactrocera minax | 108.306332 | 28.882921 |
| Bactrocera minax | 110.19516 | 28.889891 |
| Bactrocera minax | 109.811285 | 29.011727 |
| Bactrocera minax | 107.233333 | 29.033333 |
| Bactrocera minax | 114.95 | 29.05 |
| Bactrocera minax | 110.406436 | 29.098717 |
| Bactrocera minax | 113.466667 | 29.133333 |
| Bactrocera minax | 109.624679 | 29.167664 |
| Bactrocera minax | 107.836544 | 29.190575 |
| Bactrocera minax | 111.470067 | 29.243385 |
| Bactrocera minax | 111.205278 | 29.275 |
| Bactrocera minax | 110.030921 | 29.276975 |
| Bactrocera minax | 107.7 | 29.3 |
| Bactrocera minax | 107.729444 | 29.315 |
| Bactrocera minax | 107.771117 | 29.333057 |
| Bactrocera minax | 107.755556 | 29.348889 |
| Bactrocera minax | 107.881586 | 29.397551 |
| Bactrocera minax | 111.448567 | 29.516322 |
| Bactrocera minax | 108.05 | 29.533333 |
| Bactrocera minax | 111.3826 | 29.5591 |
| Bactrocera minax | 111.380008 | 29.564501 |
| Bactrocera minax | 111.843184 | 29.565177 |
| Bactrocera minax | 107.91 | 29.573333 |
| Bactrocera minax | 111.497078 | 29.575853 |
| Bactrocera minax | 111.698056 | 29.609167 |
| Bactrocera minax | 111.214481 | 29.641673 |
| Bactrocera minax | 108.083333 | 29.666667 |
| Bactrocera minax | 111.313222 | 29.695041 |
| Bactrocera minax | 112.066667 | 29.733333 |
| Bactrocera minax | 110.932805 | 29.800585 |
| Bactrocera minax | 110.93111 | 29.801606 |
| Bactrocera minax | 111.685227 | 29.838258 |
| Bactrocera minax | 107.537788 | 29.858169 |
| Bactrocera minax | 106.916667 | 29.95 |
| Bactrocera minax | 111.070833 | 29.958333 |
| Bactrocera minax | 112.070833 | 29.958333 |
| Bactrocera minax | 112.25 | 30.05 |
| Bactrocera minax | 111.656859 | 30.092015 |
| Bactrocera minax | 111.79305 | 30.130215 |
| Bactrocera minax | 111.684534 | 30.136786 |
| Bactrocera minax | 111.767431 | 30.170322 |
| Bactrocera minax | 111.729246 | 30.183667 |
| Bactrocera minax | 111.469302 | 30.198389 |
| Bactrocera minax | 111.500127 | 30.221995 |
| Bactrocera minax | 111.567789 | 30.255957 |
| Bactrocera minax | 111.49553 | 30.259051 |
| Bactrocera minax | 111.283333 | 30.3 |
| Bactrocera minax | 112.283333 | 30.3 |
| Bactrocera minax | 111.50728 | 30.303975 |
| Bactrocera minax | 111.407949 | 30.322514 |
| Bactrocera minax | 103.833333 | 30.333333 |
| Bactrocera minax | 111.5726 | 30.341957 |
| Bactrocera minax | 112.133333 | 30.35 |
| Bactrocera minax | 112.176081 | 30.438713 |
| Bactrocera minax | 111.398656 | 30.440353 |
| Bactrocera minax | 111.307134 | 30.452844 |
| Bactrocera minax | 111.224539 | 30.476188 |
| Bactrocera minax | 112.016867 | 30.483123 |
| Bactrocera minax | 111.20325 | 30.486165 |
| Bactrocera minax | 111.305102 | 30.585325 |
| Bactrocera minax | 111.124722 | 30.599444 |
| Bactrocera minax | 111.065752 | 30.606716 |
| Bactrocera minax | 108.90798 | 30.657596 |
| Bactrocera minax | 108.955198 | 30.672171 |
| Bactrocera minax | 111.439245 | 30.755657 |
| Bactrocera minax | 108.911955 | 30.792414 |
| Bactrocera minax | 110.333333 | 30.8 |
| Bactrocera minax | 108.846682 | 30.812343 |
| Bactrocera minax | 108.850142 | 30.829589 |
| Bactrocera minax | 111.076591 | 30.834978 |
| Bactrocera minax | 111.421331 | 30.858522 |
| Bactrocera minax | 108.757765 | 30.874644 |
| Bactrocera minax | 110.838628 | 30.885798 |
| Bactrocera minax | 108.861005 | 30.887665 |
| Bactrocera minax | 110.017848 | 30.948159 |
| Bactrocera minax | 110.136068 | 31.010585 |
| Bactrocera minax | 109.800256 | 31.068528 |
| Bactrocera minax | 107.033333 | 31.116667 |
| Bactrocera minax | 111.466667 | 31.166667 |
| Bactrocera minax | 108.653479 | 31.222183 |
| Bactrocera minax | 109.82665 | 31.260034 |
| Bactrocera minax | 111.533333 | 31.283333 |
| Bactrocera minax | 108.666667 | 31.35 |
| Bactrocera minax | 111.083333 | 31.35 |
| Bactrocera minax | 108.725702 | 31.377362 |
| Bactrocera minax | 105.935278 | 31.442222 |
| Bactrocera minax | 105.869641 | 31.462526 |
| Bactrocera minax | 104.526389 | 31.540556 |
| Bactrocera minax | 112.4 | 31.55 |
| Bactrocera minax | 109.15 | 31.55 |
| Bactrocera minax | 105.781447 | 31.630283 |
| Bactrocera minax | 106.167419 | 31.668879 |
| Bactrocera minax | 105.753312 | 31.710414 |
| Bactrocera minax | 104.784042 | 31.853956 |
| Bactrocera minax | 106.117855 | 32.237087 |
| Bactrocera minax | 105.233333 | 32.3 |
| Bactrocera minax | 105.291667 | 32.321667 |
| Bactrocera minax | 111.027058 | 32.534364 |
| Bactrocera minax | 110.984689 | 32.553347 |
| Bactrocera minax | 110.783333 | 32.666667 |
| Bactrocera minax | 107.470556 | 32.793889 |
| Bactrocera minax | 110.818577 | 32.840592 |
| Bactrocera minax | 110.842267 | 32.863317 |
| Bactrocera minax | 111.866667 | 32.916667 |
| Bactrocera minax | 107.281478 | 33.216976 |
| Bactrocera minax | 111.6 | 33.366667 |
| Bactrocera minax | 109.633333 | 33.383333 |
| Bactrocera minax | 109.790001 | 27.209999 |
| Bactrocera minax |  |  |
| Bactrocera minax | 110.33 | 26.4 |
| Bactrocera minax | 107.408 | 27.2917 |
| Bactrocera minax | 109.231 | 26.4564 |
| Bactrocera minax | 106.834 | 26.9139 |
| Bactrocera minax | 108.278 | 28.3267 |
| Bactrocera minax | 110.330002 | 26.4 |
| Bactrocera minax | 111.18 | 29.4 |
| Bactrocera minax | 113.959999 | 28.73 |
| Bactrocera minax | 111.480003 | 28.389999 |
| Bactrocera minax | 109.809998 | 29 |
| Bactrocera minax | 109.949997 | 28.620001 |
| Bactrocera minax | 109.459999 | 28.280001 |
| Bactrocera minax | 110.760002 | 26.540001 |
| Bactrocera minax | 111.82 | 27.209999 |
| Bactrocera minax | 110.720001 | 27.08 |
| Bactrocera minax | 109.730003 | 26.68 |
| Bactrocera minax | 109.769997 | 27.219999 |
| Bactrocera minax | 110.730003 | 26.469999 |
| Bactrocera minax | 111.059998 | 29.65 |
| Bactrocera minax | 112.440002 | 28.440001 |
| Bactrocera minax | 109.790001 | 29.01 |
| Bactrocera minax | 111.900002 | 27.25 |
| Bactrocera minax | 109.82 | 27.799999 |
| Bactrocera minax | 109.410004 | 28.620001 |
| Bactrocera minax | 110.059998 | 28.23 |
| Bactrocera minax | 104.050003 | 27.629999 |
| Bactrocera minax | 111.489998 | 28.91 |
| Bactrocera minax | 104.07 | 30.68 |
| Bactrocera minax | 104.639999 | 28.76 |
| Bactrocera minax | 104.830002 | 26.6 |
| Bactrocera minax | 104 | 30.25 |
| Bactrocera minax | 106.699997 | 27.200001 |
| Bactrocera minax | 103.050003 | 27.629999 |
| Bactrocera minax | 104.080002 | 30.68 |
| Bactrocera minax | 104.059998 | 30.68 |
| Bactrocera minax | 103.889999 | 27.75 |
| Bactrocera minax | 109.929 | 29.0048 |
| Bactrocera minax | 111.399 | 29.5858 |
| Bactrocera minax | 112.437 | 28.4391 |
| Bactrocera minax | 111.476 | 28.3873 |
| Bactrocera minax | 109.807 | 29.0023 |
| Bactrocera minax | 110.057 | 28.2341 |
| Bactrocera minax | 109.408 | 28.6154 |
| Bactrocera minax | 110.759 | 26.5387 |
| Bactrocera minax | 109.825 | 27.8036 |
| Bactrocera minax | 109.788 | 27.2104 |
| Bactrocera minax | 110.726 | 26.4652 |
| Bactrocera minax | 113.961 | 28.7303 |
| Bactrocera minax | 109.734 | 26.6774 |
| Bactrocera minax | 103.889 | 27.752 |
| Bactrocera minax | 109.365 | 32.8351 |
| Bactrocera minax | 111.175 | 29.4025 |
| Bactrocera minax | 110.721 | 27.0806 |
| Bactrocera minax | 109.951 | 28.6171 |
| Bactrocera minax | 111.825 | 27.2085 |
| Bactrocera minax | 111.065 | 29.6536 |
| Bactrocera minax | 109.787 | 29.0067 |
| Bactrocera minax | 109.458 | 28.2802 |
| Bactrocera minax | 111.896 | 27.2478 |
| Bactrocera minax | 104.046 | 27.6284 |
| Bactrocera minax | 109.769 | 27.2208 |
| Bactrocera minax | 103.048 | 27.627 |
| Bactrocera minax | 104.643 | 28.763 |
| Bactrocera minax | 111.487 | 28.91 |
| Bactrocera minax | 104.069 | 30.683 |
| Bactrocera minax | 104.064 | 30.683 |
| Bactrocera minax | 104.072 | 30.683 |
| Bactrocera minax | 104.076 | 30.683 |
| Bactrocera minax | 104.829 | 26.598 |
| Bactrocera minax | 104.075 | 30.683 |
| Bactrocera minax | 104.078 | 30.683 |
| Bactrocera minax | 104.071 | 30.683 |
| Bactrocera minax | 104.073 | 30.683 |
| Bactrocera minax | 104.067 | 30.683 |
| Bactrocera minax | 104.066 | 30.683 |
| Bactrocera minax | 104.068 | 30.683 |
| Bactrocera minax | 104.074 | 30.683 |
| Bactrocera minax | 104.07 | 30.683 |
| Bactrocera minax | 106.7 | 27.2 |
| Bactrocera minax | 104.077 | 30.683 |
| Bactrocera minax | 104.065 | 30.683 |
| Bactrocera minax | 77.5946 | 12.9716 |
| Bactrocera minax | 104.0759 | 30.6517 |
| Bactrocera minax | 90.187 | 27.0322 |
| Bactrocera minax | 88.4667 | 27.0669 |
| Bactrocera minax | 88.5122 | 27.533 |
| Bactrocera minax | 104.1954 | 35.8617 |
