# Supplementary material for: Geographical distribution of two major quarantine fruit flies (Bactrocera minax Enderlein and Bactrocera dorsalis Hendel) in Sichuan Basin based on four SDMs
Source: PeerJ. 2024 Jan 8;12:e16745. doi: 10.7717/peerj.16745 (PMC10782948; doi:10.7717/peerj.16745)
Supplement: Supplemental Information 2 [file peerj-12-16745-s002.docx]

| **Species** | **longitude** | **latitude** |
| --- | --- | --- |
| Bactrocera dorsalis | 121.018111 | 24.77875 |
| Bactrocera dorsalis | 113.908683 | 22.55467 |
| Bactrocera dorsalis | 98.862232 | 18.90024 |
| Bactrocera dorsalis | 73.770682 | 18.649178 |
| Bactrocera dorsalis | 119.412 | 32.195 |
| Bactrocera dorsalis | 120.004 | 31.435 |
| Bactrocera dorsalis | 121.49 | 31.405 |
| Bactrocera dorsalis | 119.96 | 30.049 |
| Bactrocera dorsalis | 117.227 | 31.821 |
| Bactrocera dorsalis | 114.305 | 30.593 |
| Bactrocera dorsalis | 112.939 | 28.228 |
| Bactrocera dorsalis | 106.414 | 29.806 |
| Bactrocera dorsalis | 120.93 | 24.631 |
| Bactrocera dorsalis | 109.483 | 19.5 |
| Bactrocera dorsalis | 110.198 | 20.044 |
| Bactrocera dorsalis | 108.883 | 18.75 |
| Bactrocera dorsalis | 109.414 | 18.311 |
| Bactrocera dorsalis | 110.798 | 19.543 |
| Bactrocera dorsalis | 102.893 | 23.747 |
| Bactrocera dorsalis | 100.833 | 21.983 |
| Bactrocera dorsalis | 102.833 | 24.88 |
| Bactrocera dorsalis | 98.849 | 23.495 |
| Bactrocera dorsalis | 97.852 | 24.013 |
| Bactrocera dorsalis | 104.4 | 23.614 |
| Bactrocera dorsalis | 106.9595 | 23.713 |
| Bactrocera dorsalis | 111.277 | 24.814 |
| Bactrocera dorsalis | 110.356 | 22.055 |
| Bactrocera dorsalis | 106.75 | 22.1 |
| Bactrocera dorsalis | 113.351 | 23.158 |
| Bactrocera dorsalis | 116.122 | 24.289 |
| Bactrocera dorsalis | 113.598 | 24.81 |
| Bactrocera dorsalis | 112.465 | 23.047 |
| Bactrocera dorsalis | 119.234 | 26.083 |
| Bactrocera dorsalis | 118.095 | 26.581 |
| Bactrocera dorsalis | 118.062 | 24.448 |
| Bactrocera dorsalis | 115.348 | 26.335 |
| Bactrocera dorsalis | 111.344 | 25.274 |
| Bactrocera dorsalis | 104.895 | 25.092 |
| Bactrocera dorsalis | 101.763 | 26.421 |
| Bactrocera dorsalis | 105.817 | 21.033 |
| Bactrocera dorsalis | 117.16667 | 31.91667 |
| Bactrocera dorsalis | 107.83333 | 30.08333 |
| Bactrocera dorsalis | 118 | 26.25 |
| Bactrocera dorsalis | 113.25 | 23.5 |
| Bactrocera dorsalis | 109 | 24 |
| Bactrocera dorsalis | 107 | 27 |
| Bactrocera dorsalis | 109.75 | 19.25 |
| Bactrocera dorsalis | 112.25 | 31 |
| Bactrocera dorsalis | 111.75 | 27.66667 |
| Bactrocera dorsalis | 119.83333 | 33 |
| Bactrocera dorsalis | 115.66667 | 27.66667 |
| Bactrocera dorsalis | 121.41667 | 31.16667 |
| Bactrocera dorsalis | 102.66667 | 30.5 |
| Bactrocera dorsalis | 88 | 31.66667 |
| Bactrocera dorsalis | 101.5 | 25 |
| Bactrocera dorsalis | 120 | 29.16667 |
| Bactrocera dorsalis | 114.16667 | 22.25 |
