# Supplementary material for: Geographical distribution of two major quarantine fruit flies (Bactrocera minax Enderlein and Bactrocera dorsalis Hendel) in Sichuan Basin based on four SDMs
Source: PeerJ. 2024 Jan 8;12:e16745. doi: 10.7717/peerj.16745 (PMC10782948; doi:10.7717/peerj.16745)
Supplement: Supplemental Information 3 [file peerj-12-16745-s003.docx]

| **code** | **Variables and description** | **Unit** |
| --- | --- | --- |
| Bio1 | Annual Mean Temperature | ℃ |
| Bio2 | Mean Diurnal Range(Mean of monthly (max temp - min temp) | ℃ |
| Bio3 | Isothermality (BIO2/BIO7) (* 100) | / |
| Bio4 | Temperature Seasonality (standard deviation *100) | ℃ |
| Bio5 | Max Temperature of Warmest Month | ℃ |
| Bio6 | Min Temperature of Coldest Month | ℃ |
| Bio7 | Temperature Annual Range (BIO5-BIO6) | ℃ |
| Bio8 | Mean Temperature of Wettest Quarter | ℃ |
| Bio9 | Mean Temperature of Driest Quarter | ℃ |
| Bio10 | Mean Temperature of Warmest Quarter | ℃ |
| Bio11 | Mean Temperature of Coldest Quarter | ℃ |
| Bio12 | Annual Precipitation | mm |
| Bio13 | Precipitation of Wettest Month | mm |
| Bio14 | Precipitation of Driest Month | mm |
| Bio15 | Precipitation Seasonality (Coefficient of Variation) | / |
| Bio16 | Precipitation of Wettest Quarter | mm |
| Bio17 | Precipitation of Driest Quarter | mm |
| Bio18 | Precipitation of Warmest Quarter | mm |
| Bio19 | Precipitation of Coldest Quarter | mm |
| Alt | Elevation | m |
